# Hypoxyprobe™ reveals dynamic spatial and temporal changes in hypoxia in a mouse model of endometrial breakdown and repair

**DOI:** 10.1186/s13104-016-1842-8

**Published:** 2016-01-19

**Authors:** Fiona L. Cousins, Alison A. Murray, Jessica P. Scanlon, Philippa T. K. Saunders

**Affiliations:** Medical Research Council Centre for Inflammation Research, The University of Edinburgh, 47 Little France Crescent, Edinburgh, EH16 4TJ UK; Medical Research Council Centre for Reproductive Health, The University of Edinburgh, 47 Little France Crescent, Edinburgh, EH16 4TJ UK

**Keywords:** Endometrium, Hypoxia, Menstruation, Repair, Angiogenesis

## Abstract

**Background:**

Menstruation is the culmination of a cascade of events, triggered by the withdrawal of progesterone at the end of the menstrual cycle. Initiation of tissue destruction and endometrial shedding causes spiral arteriole constriction in the functional layer of the endometrium. Upregulation of genes involved in angiogenesis and immune cell recruitment, two processes that are essential to successful repair and remodelling of the endometrium, both thought to be induced by reduced oxygen has been reported. Evidence for stabilisation/increased expression of the transcriptional regulator hypoxia inducible factor in the human endometrium at menses has been published. The current literature debates whether hypoxia plays an essential role during menstrual repair, therefore this study aims to delineate a role for hypoxia using a sensitive detection method (the Hypoxyprobe™) in combination with an established mouse model of endometrial breakdown and repair.

**Results:**

Using our mouse model of menses, during which documented breakdown and synchronous repair occurs in a 24 h timeframe, in combination with the Hypoxyprobe™ detection system, oxygen tensions within the uterus were measured. Immunostaining revealed striking spatial and temporal fluctuations in hypoxia during breakdown and showed that the epithelium is also exposed to hypoxic conditions during the repair phase. Furthermore, time-dependent changes in tissue hypoxia correlated with the regulation of mRNAs encoding for the angiogenic genes vascular endothelial growth factor and stromal derived factor (*Cxcl12*).

**Conclusions:**

Our findings are consistent with a role for focal hypoxia during endometrial breakdown in regulating gene expression during menses. These data have implications for treatment of endometrial pathologies such as heavy menstrual bleeding.

## Background

In women demise of the corpus luteum in the absence of pregnancy results in a rapid decline in peripheral progesterone that precipitates a cascade of molecular changes which induce myometrial contractions and vasoconstriction of the spiral arterioles of the endometrium. The net result of these changes is the generation of a hypoxic microenvironment in the upper, decidualised, functional layer of the endometrium which is typical of that associated with tissue necrosis and ischaemic injury [1]. Under normoxia, cellular oxygen sensors hydroxylate specific proline residues in the HIF-1α (hypoxia inducible factor-1 alpha) protein targeting it for rapid proteasome dependent degradation [2, 3]. Under hypoxic conditions, degradation of HIF-1α does not occur and the protein translocates to the nucleus where it forms heterodimeric binding complexes with HIF-β [4]. The HIF heterodimer binds to hypoxic response elements, acting as a transcription factor regulating expression of hypoxic response genes a number of which are expressed in the endometrium during menses [5–7].

HIF1α protein and its mRNA have been identified in the human endometrium, however the number of samples recovered during the menstrual phase was low with only n = 2 [5]. Previous studies using HIF expression as a surrogate for detection of hypoxia in the endometrium have yielded inconsistent results [5, 8]. As HIF is notoriously unstable it is possible that expression is lost during tissue collection and fixation or that the duration of hypoxia was too brief to stabilise enough HIF for immunohistochemical detection.

There is some disagreement in the literature as to whether hypoxia plays an essential role in regulation of tissue breakdown and initiation of repair during menses. Notably it is reported that the pattern of immunoexpression of HIF1α, HIF1β and HIF2α across the menstrual cycle is inconsistent with a role for hypoxia at menses [8]. Conversely in the same study, in vitro culture of endometrial stromal cells under hypoxic conditions resulted in an increase in intracellular HIF1α and VEGF [8] and together with data from Maybin et al. [9] would appear to support a role for hypoxia at menses.

These apparently contradictory results have prompted use of alternatives to immunohistochemistry including electron paramagnetic resonance (EPR) oximetry in combination with implanted particulate materials for in vivo measurement of oxygen tension. However, the use of these particulates does not generate images of the sample sites and successful EPR is highly dependent upon the microenvironment surrounding the particulate. A xenograft model of menstruation has utilised EPR in combination with lithium phtalocyanine (LiPc) crystals, which are sensitive to oxygen levels below 10 mmHg [10], this study reported no consistent evidence of hypoxia during menses. However, in this study the tissue was restricted to samples from the functional layer and therefore lacked the spiral arterioles of the basal layer that are thought to vasoconstrict at menstruation. An additional complication was that the model did not consider the role that the microenvironment of the flank engraftment site may play in xenograft establishment and HIF degradation during steroid induced ‘menstruation’ in the human tissue fragment.

The development of the Hypoxyprobe™ system facilitated direct visualisation of hypoxia in situ at oxygen concentrations of <1 %, irrespective of how long a cell has been under hypoxic conditions, but more importantly detection of the probe is resistant to fixation. The Hypoxyprobe™ system uses pimonidazole hydrochloride as its hypoxia marker, which forms protein adducts in hypoxic cells (pO_2_ < 10 mmHg) when administered in vivo. These protein adducts can be visualised by a specific monoclonal antibody. Detection of pimonidazole has been shown to be a reliable and direct measure of tissue hypoxia.

We hypothesised that hypoxia does occur during endometrial breakdown and that it is therefore likely to be a major player in promoting changes in gene expression that support rapid tissue repair at menses. Using the Hypoxyprobe™ in conjunction with a validated mouse model of rapid endometrial breakdown and repair revealed a spatial and temporal gradient of hypoxia in the endometrium which was associated with temporal changes in expression of angiogenic factors that are integral to restoration of a normal endometrial architecture.

## Methods

C57BL6 mice of 8–12 weeks of age were used in a previously validated protocol [11] in combination with the Hypoxyprobe™ labelling system. Experiments were performed in accordance with UK legal requirements and under licensed approval from the UK Home Office (London) following Ethical Review. Mice were given access to food and water ad libitum and housed in accordance with Home Office guidelines. Briefly, mice underwent the menses protocol as detailed previously. Mice were culled at specific time-points; 0 h (time of progesterone withdrawal, achieved by the removal of a progesterone-secreting pellet, PWD), 4 h (after PWD), 8 h (after PWD) and 24 h (after PWD). Ninety minutes prior to culling each mouse received a single intra-peritoneal injection of the pimonidazole (15 mg/ml in PBS). Uteri were dissected, fixed in 4 % neutral buffered formalin and processed into paraffin wax. Hypoxia was investigated in transverse uterine sections using standard protocols facilitating detection of pimonidazole with the Hypoxyprobe™ antibody (diluted 1/100 in normal animal serum). Hypoxia, defined as <1 % oxygen was visualised as a brown stain following immunohistochemistry by DAB chromogen detection. QRTPCR analysis was performed as described previously [11]; *Vegfa* Fwd aaaaacgaaagcgcaagaaa Rev tttctccgctctgaacaagg, *Cxcl12* Fwd ctgtgcccttcagattgtt Rev taatttcgggtcaatgcaca, *Flt1* Fwd ggcccgggatatttataagaac Rev ccatccattttaggggaagtc, *Kdr* Fwd cagtggtactggcagctagaag Rev acaagcatacgggcttgttt. Each sample was expressed relative to the endogenous control 18 s and then compared to the mean 0 h time-point sample set. Statistical analysis was performed by One Way ANOVA with Bonferroni post hoc testing where statistical significance was accepted where p < 0.05. Histograms for each gene are depicted as the mean ± the standard error of the mean.

## Results

**Fig. 1:**
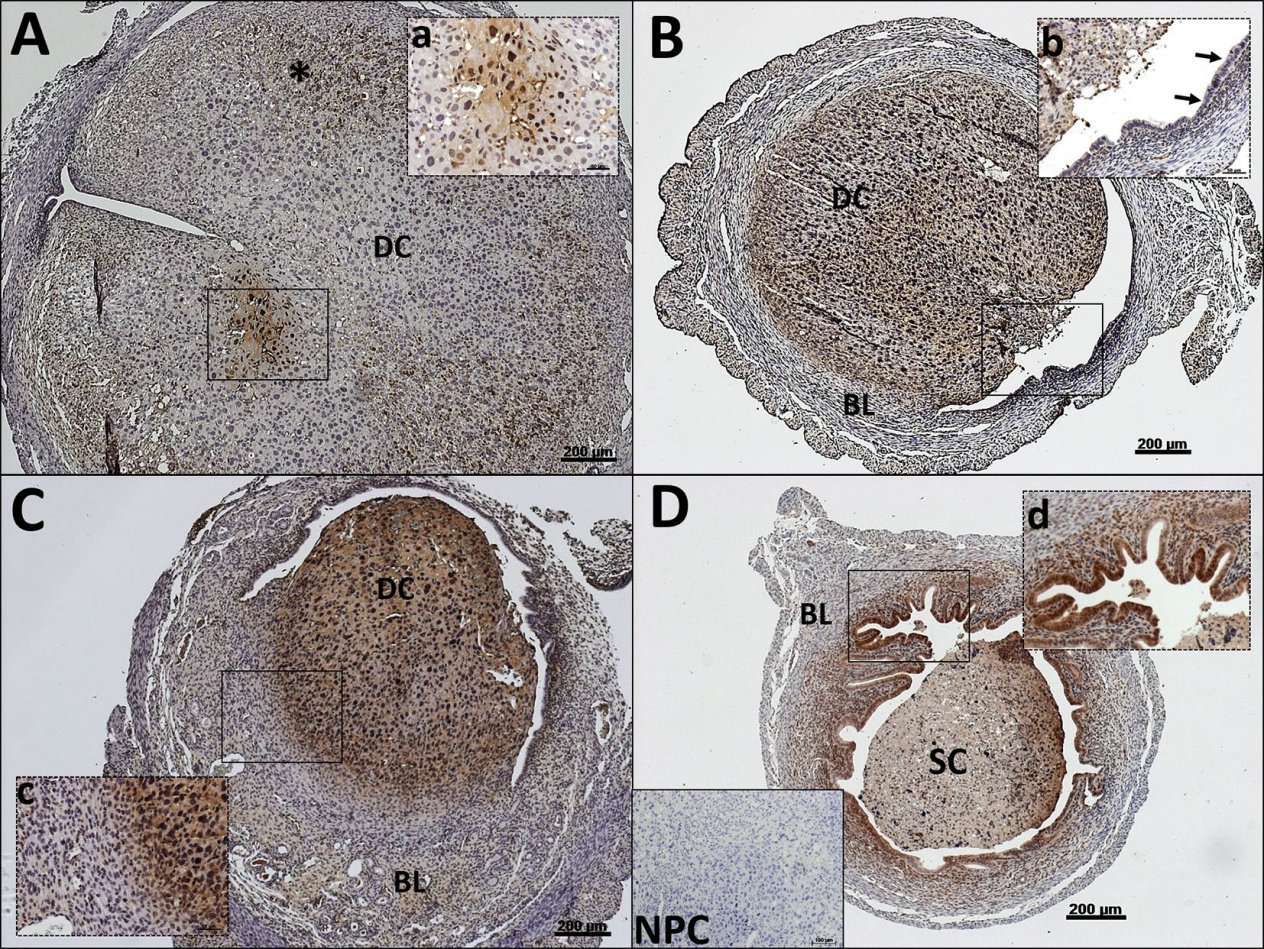
The endometrium is exposed to hypoxic conditions in a temporal and spatial manner during endometrial breakdown and repair. **A** Time of progesterone withdrawal (0 h), hypoxia is detected in the decidualised cell mass (*inset* **a**), **B** 4 h after progesterone withdrawal (PWD), the decidualised cell mass is positively stained for pimonidazole. The luminal epithelium is unstained (*inset* **b**, *arrows*), **C** The intensity of staining is stronger at 8 h after PWD. A distinct gradient is detected at the border of basal layer and the decidualised functional layer (*inset* **c**), **D** At 24 h after PWD the re-epithelialisation luminal epithelium and the adjacent stroma is immunopositive (*inset* **d**). *BL* basal layer, *DC* decidualised cells, *SC* shed cells, *asterisk* evidence of hemosiderin, *NPC* negative primary control

Immunostaining for the Hypoxyprobe™ identified spatial and temporal hypoxia in the endometrium. At time of PWD (0 h, Fig. 1A) the endometrium was largely unstained for pimonidazole, an indication of normoxia. An area in the decidua was observed to be positively stained for pimonidazole protein adducts (Fig. 1A, inset a). Hemosiderin, most commonly found in macrophages, was observed in the basal layer (Fig. 1A, asterisk). This observation is consistent with findings from Kaitu’u-Lino et al. who observed macrophages residing in the basal layer at the time of PWD [12]. Within 4 h of PWD (Fig. 1B) the decidualised cell mass was intensely stained for hypoxia, whilst the basal layer remained unstained. The luminal epithelium was negative for hypoxia (indicated by the arrows in Fig. 1B, inset b), whilst the stromal cells adjacent to the luminal epithelium were positively stained. The intensity of staining of pimonidazole adducts was stronger in the shedding decidual cell mass in tissues collected 8 h after the withdrawal of progesterone (Fig. 1C). The luminal epithelium remained unstained. The basal layer also remained normoxic, with the existence of a defined gradient in the intensity of staining in the decidualised cells and the underlying basal layer (Fig. 1C, inset c) consistent with spatial differences in hypoxia within the tissue. Within 24 h the uterine horn had regressed in size and the shed cells in the lumen were largely unstained for hypoxia (Fig. 1D). However, the luminal epithelium and the adjacent stroma were intensely stained (Fig. 1D, inset d) indicating that the endometrium is still under hypoxic conditions during endometrial repair.

**Fig. 2:**
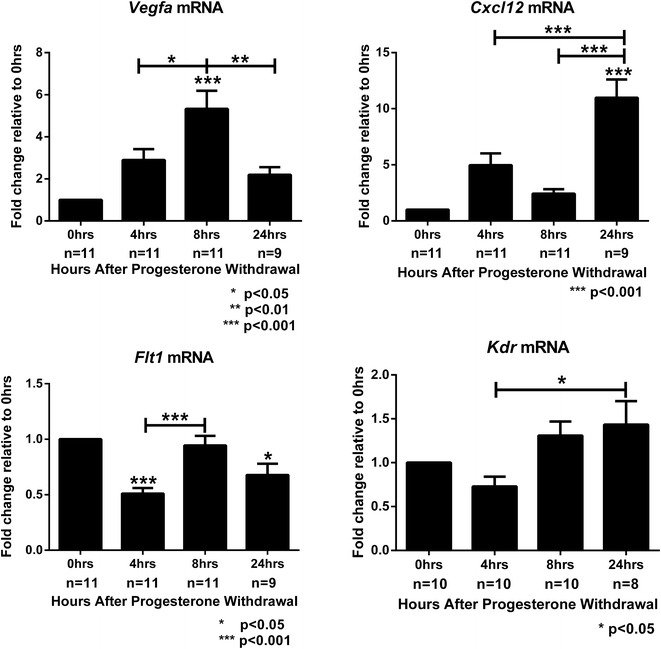
Onset of menstruation and hypoxia results in differential regulation of angiogenic genes *Vegfa, Cxcl12, Flt1* (VEGFR1) and *Kdr* (VEGFR2) All samples were normalised against the endogenous control 18 s then compared relative to the mean of 0 h time-point sample set. Statistical analysis was performed by one way ANOVA using Bonferroni post hoc testing, where statistical significance was accepted at p < 0.05. *Histograms* are depicted as the mean ± the standard* error* of the mean

It has been reported that the genes implicated in regulation of angiogenesis including *Vegfa* (vascular endothelial growth factor a) and *Cxcl12* (also known as stromal derived factor) are regulated by hypoxia in human endometrial stromal cells in vitro [9, 13]. Treatment of mice with a potent Vegf blocker (Vegf-trap) blocks neovascularisation and inhibits re-epithelialisation in endometrium of both macaque and mouse [14]. In addition, VEGF receptor 1 (VEGFR1 also known as *Flt1*) and VEGFR2 (*Kdr*) have been reported to be differentially regulated by hypoxia in a number of organs and cell types [15–17], therefore the abundance of the mRNAs encoded by these genes was investigated by qRTPCR at the same time points (Fig. 2). Concentrations of mRNAs for *Vegfa* were significantly increased at 8 h (p < 0.001) after progesterone withdrawal, consistent with intense immunostaining for hypoxia at this time-point. *Vegfa* mRNA concentrations were significantly decreased at 24 h (p < 0.01), when compared to the 8 h time-point. In contrast there was no change in mRNA concentrations for *Cxcl12* between 0 and 8 h, consistent with the tissue being under hypoxic conditions. As the tissue breaks down and the hypoxic decidualised cell mass is shed, a significant increase in *Cxcl12* was observed (p < 0.001) consistent with findings from Tsuzuki et al. [13] who observed a decrease in CXCL12 mRNA expression in human stromal cells under hypoxia in comparison to cells incubated under normoxic conditions. In contrast to findings in the brain, kidney and liver where VEGFR1 was induced by hypoxia, *Flt1* mRNA concentrations were significantly down-regulated at 4 h (p < 0.001, Fig. 2) coincident with intense staining for hypoxia, *Flt1* mRNAs were still decreased, in comparison with the 0 h time-point, at the 24 h time-point suggesting that hypoxia does regulate VEGFR1 but in a tissue dependent manner. Consistent with findings by Coudyzer et al. [10] *Kdr* mRNA concentrations did not change after PWD and the onset of hypoxia (between the 0 and 8 h time-point) supporting data from other body organs which reports that VEGFR2 is not regulated by hypoxia [15].

## Conclusions

Using the Hypoxyprobe™ in combination with a mouse model in which shedding and repair of endometrium within 24 h after progesterone withdrawal can be studied, intense immunostaining for hypoxia during breakdown and shedding of the decidual mass has been shown. Examination of tissue sections revealed that a gradient of hypoxia develops during repair of the tissue consistent with regulation of genes implicated in regulation of neo-angiogenesis and epithelial repair.

## Abbreviations

ANOVA: analysis of variance
EPR: electron paramagnetic resonance
HIF/HIF1α/β: hypoxia inducible factor/hypoxia inducible factor 1 alpha/beta
LiPc: lithium phtalocyanine crystals
pO_2_: partial pressure of oxygen
PWD: progesterone withdrawal
VEGF(R): vascular endothelial growth factor (receptor)
